# Targeting sphingosine kinase 1/2 by a novel dual inhibitor SKI-349 suppresses non-small cell lung cancer cell growth

**DOI:** 10.1038/s41419-022-05049-4

**Published:** 2022-07-12

**Authors:** Yuhang Xue, Kanqiu Jiang, Li Ou, Mingjing Shen, Yi Yang, Jingjing Lu, Weihua Xu

**Affiliations:** 1 Department of Thoracic Surgery, the Second Affiliated Hospital of Soochow University, Suzhou, China; 2 Department of Gynecology and Obstetrics, The Second Affiliated Hospital Soochow University, Suzhou, China; 3 Department of Nuclear Medicine, the Affiliated Suzhou Science & Technology Town Hospital of Nanjing Medical University, Suzhou, China; 4 Department of Radiotherapy and Oncology, Affiliated Kunshan Hospital of Jiangsu University, Kunshan, China

**Keywords:** Non-small-cell lung cancer, Targeted therapies

## Abstract

Sphingosine kinase 1 (SphK1) and sphingosine kinase (SphK2) are both important therapeutic targets of non-small cell lung cancer (NSCLC). SKI-349 is a novel, highly efficient and small molecular SphK1/2 dual inhibitor. Here in primary human NSCLC cells and immortalized cell lines, SKI-349 potently inhibited cell proliferation, cell cycle progression, migration and viability. The dual inhibitor induced mitochondrial depolarization and apoptosis activation in NSCLC cells, but it was non-cytotoxic to human lung epithelial cells. SKI-349 inhibited SphK activity and induced ceramide accumulation in primary NSCLC cells, without affecting SphK1/2 expression. SKI-349-induced NSCLC cell death was attenuated by sphingosine-1-phosphate and by the SphK activator K6PC-5, but was potentiated by the short-chain ceramide C6. Moreover, SKI-349 induced Akt-mTOR inactivation, JNK activation, and oxidative injury in primary NSCLC cells. In addition, SKI-349 decreased bromodomain-containing protein 4 (BRD4) expression and downregulated BRD4-dependent genes (*Myc*, *cyclin D1* and *Klf4*) in primary NSCLC cells. At last, SKI-349 (10 mg/kg) administration inhibited NSCLC xenograft growth in nude mice. Akt-mTOR inhibition, JNK activation, oxidative injury and BRD4 downregulation were detected in SKI-349-treated NSCLC xenograft tissues. Taken together, targeting SphK1/2 by SKI-349 potently inhibits NSCLC cell growth in vitro and in vivo.

## Introduction

Lung cancer is still a common global malignancy and a leading cause of cancer-related human mortalities [1, 2]. There are two main subtypes of lung cancer, small cell lung cancer (SCLC) and non-small cell lung cancer (NSCLC). Among them, NSCLC accounts for over 80–85% of all lung cancer [1, 2]. Metastatic, recurrent and other advanced NSCLC have very limited clinical treatment options and patients are often with extremely poor prognosis [3]. Therefore, uncovering the molecular mechanisms of NSCLC and identifying novel biomarkers could be crucial for early diagnosis and better treatment [4].

Two functional sphingosine kinase (SphK) isoforms, sphingosine kinase 1 (SphK1) and sphingosine kinase (SphK2), are conserved lipid kinases phosphorylating sphingosine to sphingosine-1-phosphate (S1P) [5–8]. Both SphK1 and SphK2 play crucial roles in tumorigenesis and progression of NSCLC and other human cancers, mainly by regulating cell proliferation and apoptosis [5–8]. Liang et al., have reported a SphK1-driven NF-κB/IL-6/STAT3/ sphingosine-1-phosphate receptor 1 (S1PR1) amplification loop, essential for colitis and colitis-associated cancer (CAC) development and progression [9]. Studies have shown that downregulation of LRIG1 (leucine-rich repeat and immuno*g*lobulin-like domain containing) enhanced EGFR-MAPK-SPHK1 signaling and extracellular matrix remodeling, promoting malignant behaviors of cancer cells [9, 10].

Song et al., have shown that SphK1 expression is markedly increased in NSCLC, correlating with tumor progression and poor survival of NSCLC patients [11]. Conversely, genetic silencing or pharmacological inactivation of SphK1 robustly enhanced chemotherapeutics-induced apoptosis in NSCLC cells [11]. Zhu et al., found that SphK1 activated Akt signaling and enhanced NSCLC cell invasion and migration [12]. Ma et al., reported that SphK1 overexpression activated signal transducer and activator of transcription 3 (STAT3) signaling and promoted NSCLC cell proliferation and migration [13]. Insulin-like growth factor-1 (IGF-1) increased SphK1 expression and activation, promoting NSCLC epithelial-mesenchymal transition (EMT), cell migration and resistance of paclitaxel [14].

Wang et al., have reported that SphK2 overexpression in NSCLC is correlated with lower overall survival (OS) and disease-free survival [15]. Liu et al., have also demonstrated that SphK2 is overexpressed in NSCLC, and SphK2 knockdown inhibited proliferation and enhanced gefitinib sensitivity in NSCLC cells [16]. SphK2 inhibition, by a small molecule inhibitor ABC294640, enhanced TRAIL (tumor necrosis factor-related apoptosis-inducing ligand)-induced antitumor activity in NSCLC cells [17]. Therefore, targeting SphK1 and SphK2 could result in promising anti-NSCLC cell activity.

SKI-178, or 3-(4-methoxyphenyl)-1H-pyrazole-5-carboxylic acid, 2-[1-(3,4-dimethoxyphenyl)ethylidene]hydrazide, is a potent SphK1 and SphK2 dual inhibitor [18, 19]. It has displayed significant cytotoxic and pro-apoptotic activity in acute myeloid leukemia (AML) cells [18, 19]. Using the structure guided approach, Hengsta and colleagues modified the linker region between the substituted phenyl rings of SKI-178 and developed a SKI-178 congener, namely SKI-349 [20]. As compared to SKI-178, SKI-349 showed log-fold enhancements in both SphK inhibition and cytotoxic potency against AML cells [20]. Considering that SphK1 and SphK2 are both important therapeutic targets of NSCLC and SKI-349 is a potent SphK1/2 dual inhibitor, the current study explored the potential anti-cancer activity and the possible underlying mechanisms of SKI-349 in NSCLC cells.

## Materials and methods

### Chemicals and reagents

The fluorescence dyes utilized in the study were described previously [21, 22]. Annexin V-propidium iodide (PI) FACS (fluorescence-activated cell sorting) kit was purchased from Thermo-Fisher Invitrogen Co. (Shanghai, China). Antibodies for SphK2 (#32346), BRD4 (bromodomain-containing protein 4, #13440), p-JNK1/2 (Thr183/Tyr185, #4671) and JNK1 antibody (#3708) were purchased from Cell Signaling Technologies (Beverly, MA). Antibodies for tumor necrosis factor (TNF)-related apoptosis-inducing ligand (TRAIL, #3219), cytochrome c (Cyto-C, #4280), cathepsin B (#31718), cathepsin D (#69854), Bax (#89477) were from Signaling Tech. The anti-tBid was provided by Abcam (ab10640). Other antibodies in this study were reported previously [21, 22]. K6PC-5 was provided by Dr. Liu at Jiangsu University [23]. Sphingosine-1-phosphate (S1P) was described previously [21]. SKI-349, (4-Amino-2-((4-methoxyphenyl)amino)thiazol-5-yl)(3,4-dimethoxyphenyl)methanone, was synthesized by Shanghai Ruilu Biotech. C6 ceramide was provided by Dr. Chen [24, 25]. The SphK1 inhibitor PF-543, BML-258 and FTY720, the SphK2 specific inhibitor ABC294640 were purchased from Selleck (Shanghai, China). N-acetylcysteine (NAC) and the JNK inhibitor SP600125 were purchased from Sigma-Aldrich (St. Louis, MO). The cell culture reagents were all provide by Hyclone Co. (Logan, UT). For in vitro experiments, all inhibitors or reagents were first dissolved in DMSO (except for NAC) to make stock solutions, which were then added to cell medium with the final DMSO concentration <0.1% (vehicle). NAC was dissolved in PBS. The concentrations of the SphK1 inhibitor PF-543 BML-258 and FTY720 [26, 27] or the SphK2 specific inhibitor ABC294640 [17, 24, 28, 29], utilized at 10–25 μM, were chosen based on the literatures. NAC was dissolved in PBS. In the pre-experiments, we tested SKI-349 concentrations starting from 0.001–0.1 μM in different human NSCLC cells. None of these lower concentrations showed any significant anti-proliferative, cytotoxic and pro-apoptotic activities against the NSCLC cells.

### Cell culture

The immortalized NSCLC cell lines, A549 and NCI-H1944, the patient-derived primary human NSCLC cells, pNSCLC-1, pNSCLC-2 and pNSCLC-3 (derived from three different written-informed consent patients), as well as the BEAS-2B lung (bronchial) epithelial cells and the primary human lung epithelial cells were described in our previous studies [21, 22]. A549, NCI-H1944 and BEAS-2B cells were maintained in DMEM medium plus 8–10% FBS. The primary NSCLC cells and primary human lung epithelial cells were maintained in high glucose DMEM medium with 12% FBS plus different growth factors [21]. The gene-alteration profiles of A549 cells were reported previously [30]. NCI-H1944 cell line has mutations in *KRAS* and *ATR*. All three primary NSCLC cells *PTEN* depletion, *PI3KCA* and *KRAS* mutations. The protocols were in according to the principles of Declaration of Helsinki and were approved by the Ethics Committee of Soochow University (SDM2021-1103). Mycoplasma and microbial contamination examination were checked every month, with cell doubling time, colony forming efficiency, and morphology verified routinely.

### Cellular function studies

Normal distribution was assumed for all endpoints. For in vitro experiments, the estimated sample sizes were exceeded to ensure enough power and using standard deviations (SD). In addition, in vitro experiments were repeated in different NSCLC cells to enhance the power and the confidence of the result outcomes. Each in vitro cellular experiments were repeated five times. NSCLC cells or the epithelial cells were distributed into 96-well/12-well/6-well plates or tissue culturing slides at 70–80% confluence and treated with SKI-349 (at the applied concentrations) or the vehicle control. Cells were then maintained in the conditional medium for indicated time periods and were subject to different cellular functional assays. Cell counting kit-8 (CCK-8) viability assay, colony formation, propidium iodide (PI)-FACS, cell proliferation detection by measuring nuclear 5-ethynyl-2'-deoxyuridine (EdU) staining, BrdU incorporation ELISA assay, the in vitro cell migration/invasion by the “Transwell”/“Matrigel Transwell” assays, cell apoptosis detection by measuring the nuclear terminal deoxynucleotidyl transferase (TdT) dUTP nick-end labeling (TUNEL) staining and Annexin V-PI FACS, the caspase-3 and caspase-7 activity assays, trypan blue-staining assay of cell death, mitochondrial depolarization detection by measuring JC-1 green monomer intensity, reactive oxygen species (ROS) detection by the CellROX dye assay, lipid peroxidation detection by the thiobarbituric acid reactive substances (TBAR) activity assay were described in detail in our previous studies [21, 22, 31, 32]. Assays of the SphK activity and ceramide contents were described previously as well [21]. For “Transwell” studies, cells with SKI-349 treatment were allowed to migrate for only 24 h, and no significant cytotoxicity was yet detected.

### Gene and protein expression detection

The detailed protocols of Western blotting, quantitative real time-PCR (qRT-PCR), and data quantification were described in our previously studies [21, 22]. The verified mRNA primers were provided by Genechem (Shanghai, China). The uncroppred Western blotting images were presented in Fig. S1.

### Constitutively-active mutant Akt1

As reported [21], the recombinant constitutively-active Akt1 (caAkt1, S473D) adenoviral construct was transduced to pNSCLC-1 cells, and stable cells established by FACS sorting and selection. caAkt1 expression in the stable cells was verified by Western blotting.

### BRD4 overexpression

A BRD4 expression GV248 lentiviral construct was provided by Dr. Zheng [33] and was transduced to primary human NSCLC cells. Puromycin was then added to select stable cells for another 24 h. Expression of BRD4 was verified by Western blotting.

### SphK1/2 dual silencing

The SphK1 shRNA lentiviral particles and SphK2 shRNA lentiviral particles, both from Santa Cruz Biotech (Santa Cruz, CA), were together added to primary human NSCLC cells for 36 h. Puromycin was then added to select stable cells for another 24 h. Expression of SphK1/2 in the stable cells was tested by Western blotting.

### Tumor xenograft studies

The nude mice (18.5–19.0 g, 5–6 week old, half male hale female) were maintained under the Animal Facility of Soochow University. pNSCLC-1 cells (6 × 10^6^ cells per mouse) were subcutaneously (*s.c*.) injected to the flanks of the nude mice. The patient-derived xenograft (PDX) NSCLC model was established by subcutaneous injection of pNSCLC-1 primary cells (6 × 10^6^ for each mouse) to the nude mice. pNSCLC-1 xenografts were formed 20 days following initial cell injection, with tumor volume close to 100 mm^3^ (“Day-0”). pNSCLC-1 xenograft-bearing nude mice were then intraperitoneally injected with SKI-349 (at 10 mg/kg body weight, every other day, for seven times/14 days [18]) or the vehicle control (“Veh”, [18]). The tumor volumes were calculated using the described formula [21] and recorded weekly from “Day-0” to “Day-42”. For animal *xenograft* studies, power and sample sizes were estimated using empirical data from extensive experience in our lab for the expected SD and were supported by the current sample size calculators. All animal procedures were approved by IACUC (institutional animal care and use committee) and Ethic Committee of Soochow University (SDM2021-1103).

### Statistical analysis

Data were always with normal distribution and were presented as mean ± SD. The one-way analysis of variance (ANOVA) plus Tukey’s multiple comparison test (GraphPad Prism 5.01) were utilized for the comparison of multiple groups. For comparison between two groups, the Student t test (Excel 2007) was utilized. IC-50 was calculated by nonlinear regression analysis using GraphPad Prism 5.01. *P*-values < 0.05 were statistically significant.

## Results

### SKI-349 potently inhibits NSCLC cell progression in vitro

**Fig. 1:**
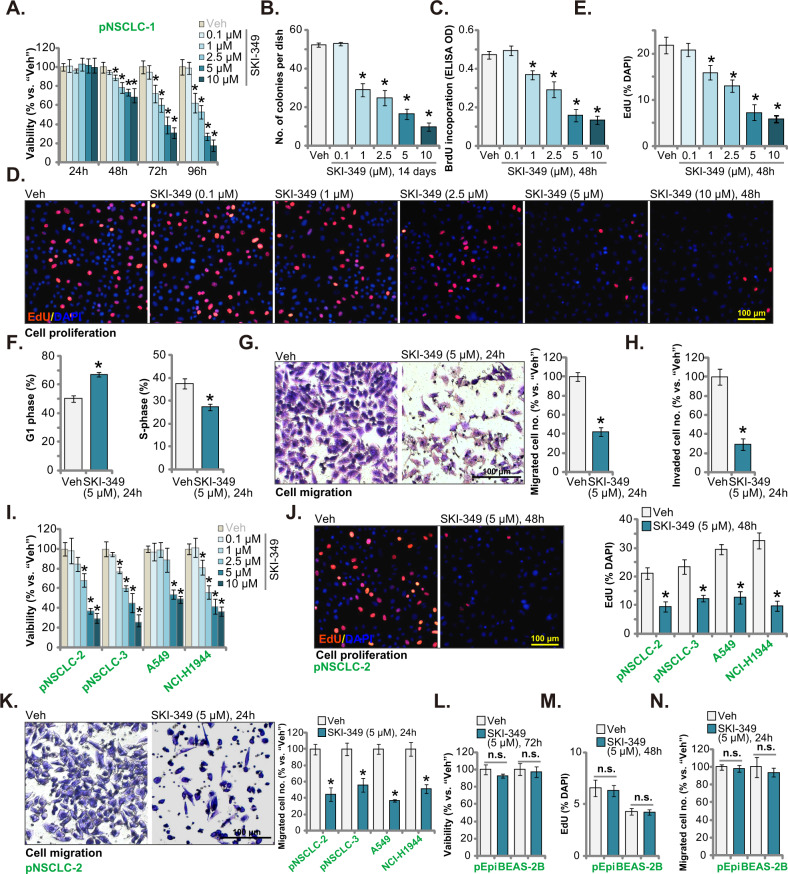
SKI-349 potently inhibits NSCLC cell progression in vitro. The patient-derived primary NSCLC cells, pNSCLC-1, were treated with SKI-349 at the applied concentration or the vehicle control (“Veh”, 0.1% DMSO), and cells were further cultivated for designated time; Cell viability (by measuring CCK-8 OD, **A**), colony number (**B**) and cell proliferation (by testing BrdU incorporation and EdU-positive nuclei percentage, **C**–**E**) as well as cell cycle distributions (**F**), in vitro cell migration and invasion (**G**, **H**) were examined by the designated assays. The patient-derived primary NSCLC cells (pNSCLC-2 and pNSCLC-3), the immortalized cell lines (A549 and NCI-H1944), the BEAS-2B lung epithelial cells or the primary human lung epithelial cells (pEpi) were treated with SKI-349 (5 μM, except for **I**) or the vehicle control (“Veh”, 0.1% DMSO), and cells were further cultivated for designated time; Cell viability (**I** and **L**), proliferation (by measuring the EdU-positively-stained nuclei percentage, **J** and **M**) and in vitro cell migration (“Transwell” assays, **K** and **N**) were tested. Data were presented as mean ± SD (*n* = 5). *P < 0.05 versus “Veh” treatment. “n.s.” stands for non-statistical difference (*P* > 0.05) (**L**–**N**). Experiments in this figure were repeated five times, and similar results were obtained. Scale bar = 100 μm.

SKI-349 efficiently decreased pNSCLC-1 cell viability in a concentration-dependent manner (Fig. 1A). At 1–10 μM, SKI-349-induced viability (CCK-8 OD) reduction was significant (Fig. 1A). The SphK1/2 dual inhibitor displayed a time-dependent response as well in decreasing pNSCLC-1 cell viability, as it required at least 48 h to produce significant viability reduction (Fig. 1A), which was lasted for at least 96 h (Fig. 1A). Figure 1B demonstrated that SKI-349 decreased the number of viable pNSCLC-1 cell colonies and it was significant at 1–10 μM (Fig. 1B).

SKI-349, at 1–10 μM, potently inhibited BrdU incorporation in pNSCLC-1 cells, suggesting its anti-proliferative activity (Fig. 1C). Moreover, the percentage of EdU-positively stained nuclei was significantly decreased in SKI-349 (1–10 μM)-treated pNSCLC-1 cells (Fig. 1D, E), further supporting the anti-proliferative activity by the dual inhibitor. The results of these titration experiments (Fig. 1A–E) showed that 5 μM of SKI-349 induced robust anti-cancer activity in NSCLC cells, and this concentration was close to IC-50 (the concentration resulting in 50% CCK-8 OD reduction), this concentration was selected for the following studies.

Next we tested whether the dual inhibitor could affect cell cycle progression. Following SKI-349 (5 μM) treatment, the G1-phase pNSCLC-1 cell percentage was significantly increased, whereas the S-phase cell percentage was decreased (Fig. 1F). These results implied that the dual inhibitor induced G1-S arrest in primary NSCLC cells. Furthermore, SKI-349 (5 μM, for 24 h) potently inhibited pNSCLC-1 cell in vitro migration and invasion (Fig. 1G–H).

Experiments were carried out to further study the potential activity of SKI-349 in other NSCLC cells. The primary NSCLC cells deriving from two other primary patients (pNSCLC-2 and pNSCLC-3, see our previous study [21]) and the immortalized NSCLC cell lines (A549 and NCI-H1944) were subject to SKI-349 treatment. The CCK-8 viability assay results showed that SKI-349, in a dose-dependent manner, deceased viability in the tested primary and immortalized human NSCLC cells (Fig. 1I). The IC-50 was again close to 5 μM in the tested NSCLC cells (Fig. 1I). Moreover, SKI-349 (5 μM) inhibited cell proliferation (EdU-positive nuclei percentage reduction, Fig. 1J) and migration (“Transwell” assays, Fig. 1K). These results clearly supported the anti-NSCLC cell activity by the SphK1/2 dual inhibitor. We also tested the potential effect of SKI-349 on the non-cancerous normal epithelial cells. In BEAS-2B lung epithelial cells and primary human lung epithelial cells (“pEpi”) [21], SKI-349 (5 μM) treatment failed to exert significant inhibition on cell viability (CCK-8 OD, Fig. 1L), proliferation (EdU-positive nuclei percentage, Fig. 1M) and migration (Fig. 1N). These results implied a cancer cell-specific effect by the dual inhibitor.

### SKI-349 provokes apoptosis in NSCLC cells

**Fig. 2:**
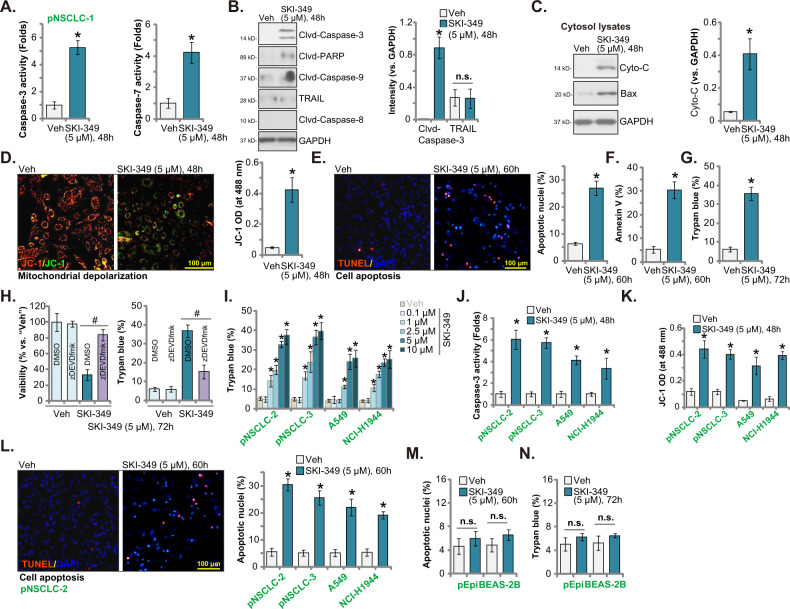
SKI-349 provokes apoptosis in NSCLC cells. The patient-derived primary human NSCLC cells, pNSCLC-1, were treated with SKI-349 (5 μM) or the vehicle control (“Veh”, 0.1% DMSO), and cells were further cultivated for designated time; The relative caspase-3 and caspase-7 activities (**A**), expression of the apoptosis-associated proteins (**B**, **C**) and depolarization of mitochondria (by measuring JC-1 green monomer intensity, (**D**)) were tested. Cell apoptosis was tested by measuring TUNEL-positively stained nuclei percentage (**E**) and Annexin-V positively gated cell percentage (**F**). Cell death was tested by measuring the ratio of Trypan blue positively-stained cells (**G**). pNSCLC-1 cells were pretreated with z-DEVD-fmk (45 μM, 30 min pretreatment) or vehicle control (0.1% DMSO), followed by SKI-349 (5 μM) stimulation and cultivated for additional 72 h, cell viability (by measuring CCK-8 OD) and death (by measuring Trypan blue positively-stained cells) were tested (**H**). The patient-derived primary NSCLC cells (pNSCLC-2 and pNSCLC-3), the immortalized cell lines (A549 and NCI-H1944), the BEAS-2B lung epithelial cells or the primary human lung epithelial cells (pEpi) were treated with SKI-349 (5 μM, expect for (**I**)) or the vehicle control (“Veh”, 0.1% DMSO), and cells were cultivated for designated time; cell death (**I** and **N**), relative caspase-3 activity (**J**), depolarization of mitochondria (**K**), cell apoptosis (**L**, **M**) were tested using the described methods. Data were presented as mean ± SD (*n* = 5). *P < 0.05 versus “Veh” treatment. ^#^*P* < 0.05 (**H**). “n.s.” stands for non-statistical difference (*P* > 0.05, **B**, **M**, **N**). Experiments in this figure were repeated five times, and similar results were obtained. Scale bar = 100 μm.

SphK inhibition can induce significant apoptosis activation in NSCLC cells [11, 13, 16, 17]. Next, we tested the potential effect of SKI-349 on cell apoptosis. Treatment with SKI-349 (5 μM, 48 h) robustly increased the caspae-3 activity and the caspase-7 activity in pNSCLC-1 primary cells (Fig. 2 A). Figure 2B confirmed that cleavages of caspae-3, caspase-9 and PARP [poly(ADP-ribose) polymerase] were increased in SKI-349-treated pNSCLC-1 cells. SKI-349 failed to significantly increase TRAIL expression and caspase-8 cleavage in pNSCLC-1 cells (Fig. 2B). Insertion of homo/hetero-oligomerized Bax and Bak into the mitochondrial outer membrane led to pore formation, membrane permeabilization, and depolarization of the mitochondria, which will cause Cyto-C release and mitochondrial apoptosis cascade activation [34, 35]. We found that the cytosol cytochrome c release and Bax expression were significantly increased in pNSCLC-1 cells after SKI-349 treatment (Fig. 2C). The dual inhibitor also induced significant mitochondrial depolarization, which was evidenced by the accumulation of JC-1 green monomer (Fig. 2D).

Nuclear TUNEL staining assay results supported that SKI-349 induced apoptosis activation in pNSCLC-1 cells, as the TUNEL-positively stained nuclei percentage was significantly increased after SKI-349 (5 μM, 72 h) treatment (Fig. 2E). Moreover the number of Annexin-V positively-stained pNSCLC-1 cell was increased after SKI-349 stimulation (Fig. 2F), again supporting apoptosis activation. The dual inhibitor induced significant pNSCLC-1 cell death, as the Trypan blue-positive cell percentage was dramatically increased (Fig. 2G).

Next, a pan-caspase inhibitor z-VAD-fmk [36–38] was utilized to block apoptosis activation. SKI-349 (5 μM, 72 h)-induced viability (CCK-8 OD) reduction (Fig. 2H) and cell death (tested by the increased number of Trypan blue-positive cells, Fig. 2H) were largely alleviated by z-VAD-fmk. These results implied that apoptosis activation should be the primary cause of SKI-349-induced cytotoxicity in pNSCLC-1 cells.

In other primary NSCLC cells (pNSCLC-2 and pNSCLC-3) and immortalized lines (A549 and NCI-H1944), the SphK1/2 dual blocker dose-dependently induced cell death in the primary and immortalized NSCLC cells, evidenced by the results from the Trypan blue-staining assays (Fig. 2I). Moreover, SKI-349 (5 μM) increased caspase-3 activity (Fig. 2J) and induced mitochondrial depolarization (JC-1 green monomer intensity increasing, Fig. 2K). The dual inhibitor induced apoptosis activation (tested by the TUNEL-positive nuclei ratio increase, Fig. 2L). In BEAS-2B cells and pEpi cells [21], SKI-349 (5 μM) treatment failed to induce significant cell apoptosis and death (Fig. 2M, N).

### SKI-349 inactivates SphK in NSCLC cells

**Fig. 3:**
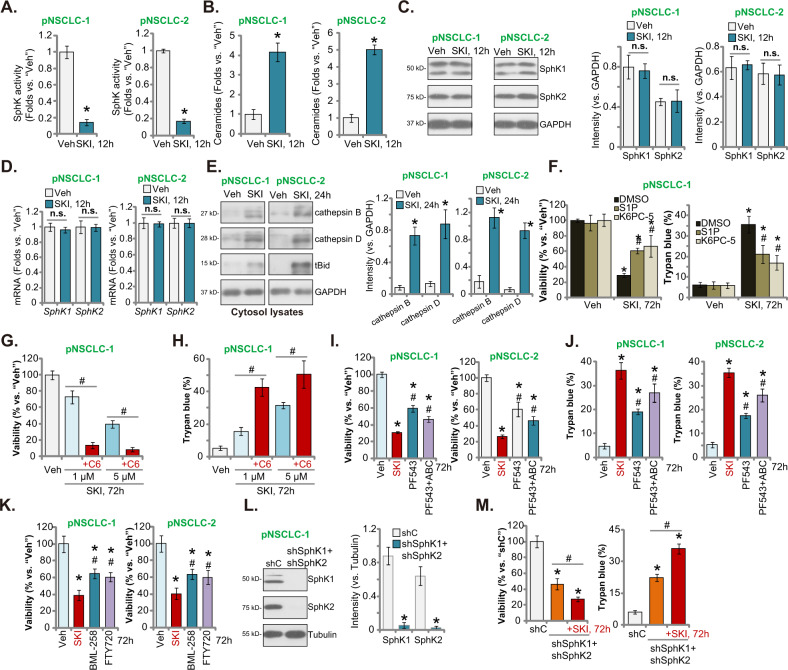
SKI-349 inactivates SphK in NSCLC cells. The patient-derived primary NSCLC cells (pNSCLC-1 and pNSCLC-2) were treated with SKI-349 (“SKI”, 5 μM) or the vehicle control (“Veh”, 0.1% DMSO), cells were further cultivated for 12 h; The relative SphK activity (**A**), cellular ceramide contents (**B**), and expression of listed proteins were tested (**C**–**E**). pNSCLC-1 primary cells were pretreated for 45 min with sphingosine-1-phosphate (S1P, 15 μM), K6PC-5 (15 μM) or 0.1% DMSO, followed by SKI-349 (“SKI”, 5 μM) stimulation and cells were further cultivated for 72 h; Cell viability (by measuring CCK-8 OD) and death (by measuring Trypan blue positively-stained cell ratio) were tested (**F**). pNSCLC-1 primary cells were treated with SKI-349 (1 or 5 μM), together with or without the short-chain C6 ceramide (10 μg/mL), and cells were further cultivated for 72 h, and cell viability (**G**) and cell death (**H**) were tested. pNSCLC-1 and pNSCLC-2 cells were treated with SKI-349 (“SKI”, 5 μM), PF-543 (25 μM), PF-543 (25 μM) plus ABC294640 (25 μM, “PF543 + ABC”), BML-258 (10 μM) or FTY720 (10 μM), or the vehicle control (“Veh”, 0.1% DMSO) for 72 h; Cell viability and death were tested (**I**–**K**). Expression of listed proteins in stable pNSCLC-1 cells expressing the scramble control shRNA (“shC”) or SphK1/2 shRNA lentiviral particles (“shSphK1 + shSphK2”) was shown (**L**); The “shSphK1 + shSphK2” pNSCLC-1 cells were further treated with or without SKI-349 (“SKI”, 5 μM) for 72 h, and cell viability (CCK-8 OD) and death (Trypan blue ratio) were tested (**M**). Data were presented as mean ± SD (*n* = 5). *P < 0.05 versus “Veh” treatment or shC treatment. ^#^*P* < 0.05 versus DMSO pretreatment (**F**). ^#^*P* < 0.05 (**G**, **H** and **M**). ^#^*P* < 0.05 versus SKI-349 treatment (**I**–**K**). “n.s.” stands for non-statistical difference (*P* > 0.05). Experiments in this figure were repeated five times, and similar results were obtained.

SKI-349 is a novel SphK1/2 dual inhibitor [20], we next analyzed its potential effect on SphK activity and SphK1/2 expression in NSCLC cells. Figure 3A demonstrated that SKI-349 (“SKI”, 5 μM) resulted in robust SphK activity reduction in patient-derived pNSCLC-1 and pNSCLC-2 cells. Consequently, pro-apoptotic total ceramide levels [39–41] were significantly increased (Fig. 3B). Notably, SphK1 and SphK2 protein and mRNA expressions were not significantly altered by the SKI-349 treatment in pNSCLC-1 and pNSCLC-2 primary cells (Fig. 3C, D). Increased ceramide could induce lysosomal release of cathepsin B and cathepsin D to cytosol, thereby induce Bid cleavage and Bax activation (Fig. 3E) [30, 42, 43]. We found that SKI-349 possibly activated cathepsin B and cathepsin D, as levels of cytosol cathepsin B and cathepsin D were both significantly increased in SKI-349-treated primary NSCLC cells. Moreover, tBid levels were increased as well (Fig. 3E).

To explore the link between SKI-349-induced SphK inactivation and NSCLC cell death, we exogenously added S1P [7, 8, 44, 45] and the SphK activator K6PC-5 [23, 46–48]. In pNSCLC-1 cells, SKI-349 (“SKI”, 5 μM, 72 h)-induced viability reduction and cell death (Fig. 3F) were ameliorated by S1P and K6PC-5. On the contrast, a short-chain C6 ceramide [25, 49–51] potentiated SKI-349 (1 and 5 μM, 72 h)-induced cytotoxicity in pNSCLC-1 cells (Fig. 3G, H). These results supported that SphK inactivation is important for SKI-349-induced NSCLC cell death.

We also compared the the activity of SKI-349 with other known SphK inhibitors, including the SphK1 inhibitor PF-543 [26, 27] and the SphK2 specific inhibitor ABC294640 [17, 24, 28, 29]. In pNSCLC-1 and pNSCLC-2 primary cells, SKI-349-induced viability reduction (Fig. 3I) and cell death (Fig. 3J) were more potent than PF-543 single treatment or plus ABC294640 co-administration (“PF543 + ABC”). In addition, SKI-349-induced cytotoxicity against primary NSCLC cells was more potent than other known SphK1 inhibitors, including BML-258 [52] and FTY720 (Fig. 3K) [53, 54]. These results suggested that inhibition of SphK1 or SphK2 each could result in NSCLC cell death. Yet SphK1 and SphK2 dual inhibition by SKI-349 shall result in even more stronger anti-NSCLC cell effect.

To silence SphK1 and SphK2, the SphK1 shRNA lentiviral particles and the SphK2 shRNA lentiviral particles were added together to pNSCLC-1 cells, and stable cells were established after puromycin selection. As shown, SphK1 and SphK2 were silenced in the stable cells (“shSphK1 + shSphK2”) (Fig. 3L). Co-silencing SphK1 and SphK2 inhibited cell viability and induced cell death (Fig. 3M) in pNSCLC-1 cells. Significantly, adding SKI-349 could further induce cytotoxicity in SphK1/2-silenced cells (Fig. 3M). These results supported that SphK1/2-indepednent mechanisms might also participate in SKI-349-induced killing of NSCLC cells.

### SKI-349 induces Akt-mTOR inhibition, JNK activation, ROS production and oxidative injury in primary human NSCLC cells

**Fig. 4:**
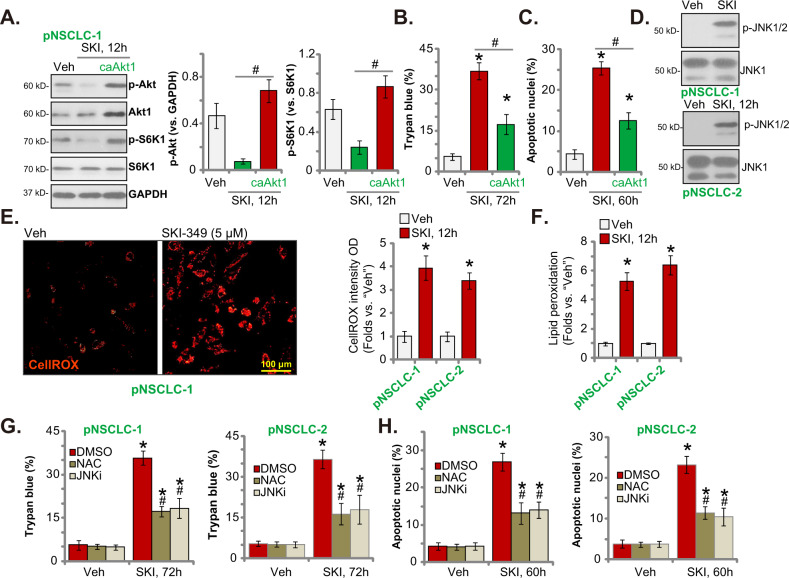
SKI-349 induces Akt-mTOR inactivation, JNK activation, ROS production and oxidative injury in primary NSCLC cells. The patient-derived pNSCLC-1 primary cells, with or without the constitutively-active S473D mutant Akt1 (caAkt1), were treated with SKI-349 (“SKI”, 5 μM), control cells were treated with the vehicle control (“Veh”, 0.1% DMSO) and cells were cultivated for designated time, expression of listed proteins was shown (**A**); Cell death (by measuring Trypan blue positively-stained cell percentage, (**B**)) and apoptosis (by measuring TUNEL-positively stained nuclei percentage, **C**) were tested. pNSCLC-1 and pNSCLC-2 cells were treated with SKI-349 (“SKI”, 5 μM) or the vehicle control (“Veh”, 0.1% DMSO), and cells were further cultivated for designated time; Expression of listed proteins (**D**), ROS contents (by measuring CellROX intensity (**E**)) and lipid peroxidation (by measuring TBAR activity, (**F**)) were tested. pNSCLC-1 and pNSCLC-2 cells were pretreated for 45 min with the antioxidant NAC (n-acetyl-L-cysteine 500 μM), the JNK inhibitor SP600125 (JNKi, 10 μM) or 0.1% DMSO, followed by SKI-349 (“SKI”, 5 μM) stimulation and cells were further cultivated for 72 h; Cell death (**G**) and apoptosis (**H**) were tested. Data were presented as mean ± SD (*n* = 5). *P < 0.05 versus “Veh” treatment. ^#^*P* < 0.05 (**A**–**C**). ^#^*P* < 0.05 versus “DMSO^”^ group (**G**, **H**). Experiments in this figure were repeated five times, and similar results were obtained. Scale bar = 100 μm (**E**).

Following SphK inactivation, S1P depletion and ceramide accumulation could result in Akt-mTOR (mammalian target of rapamycin) inactivation through multiple mechanisms [55–57]. Figure 4A showed that phosphorylations of Akt (at the Ser-473 residue) and S6K1 (at the Thr-389 residue) were largely inhibited after SKI-349 treatment (5 μM, 12 h) in pNSCLC-1 cells. Contrarily, ectopic expression of a constitutively-active S473D mutant Akt1 (caAkt1) [21] restored Akt and S6K1 phosphorylations in SKI-349-treated pNSCLC-1 cells (Fig. 4A). caAkt1 attenuated SKI-349-induced pNSCLC-1 cell death (Fig. 4B) and apoptosis (by measuring the TUNEL-positively stained nuclei percentage, Fig. 4C). These results implied that Akt-mTOR inhibition was important for SKI-349-induced NSCLC cell death.

Another important mechanism responsible for ceramide-induced apoptosis is through initiating JNK activation [58–60]. In Fig. 4D SKI-349 provoked JNK activation by inducing JNK1/2 phosphorylation in pNSCLC-1 cells. Moreover, the dual inhibitor induced oxidative injury and increased CellROX intensity in pNSCLC-1 and pNSCLC-2 cells (Fig. 4E). In addition, increased TBAR activity, indicating lipid peroxidation, was detected in primary NSCLC cells after SKI-349 treatment (Fig. 4F). Significantly, the JNK inhibitor SP600125 or the antioxidant NAC both alleviated SKI-349-induced pNSCLC-1 cell death (Fig. 4G) and apoptosis (Fig. 4H) in NSCLC cells. Therefore, SKI-349 induced JNK activation, ROS production and oxidative injury in NSCLC cells.

### SKI-349 silences BRD4 cascade in primary NSCLC cells

**Fig. 5:**
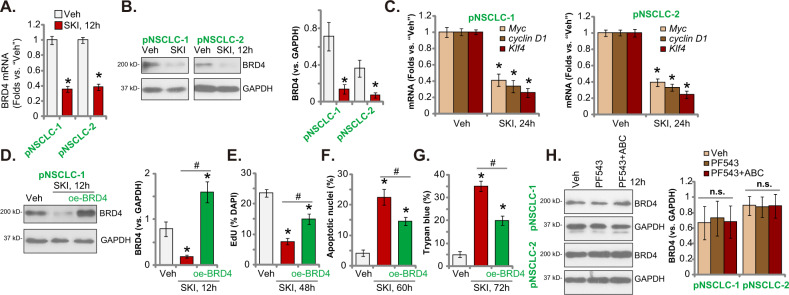
SKI-349 silences BRD4 cascade in primary NSCLC cells. The patient-derived pNSCLC-1 and pNSCLC-2 cells were treated with SKI-349 (5 μM) or the vehicle control (“Veh”, 0.1% DMSO), cells were further cultivated for designated time; Expression of listed genes and proteins were shown (**A**–**C**). pNSCLC-1 cells, with or without the lentiviral BRD4-expressing construct (“oe-BRD4”), were treated with SKI-349 (5 μM), control cells were treated with the vehicle control (“Veh”, 0.1% DMSO) and cells were cultivated for designated time, expression of listed proteins was shown (**D**); Cell proliferation (by measuring EdU-positively stained nuclei percentage, (**E**)), death (by measuring Trypan blue-positive cell percentage, (**F**)) and apoptosis (by measuring TUNEL-positively stained nuclei percentage, (**G**)) were tested. pNSCLC-1 cells were treated with PF-543 (25 μM), PF-543 (25 μM) plus ABC294640 (25 μM, “PF543 + ABC”), or the vehicle control (“Veh”, 0.1% DMSO) for 12 h, expression of listed proteins was shown (**H**). Data were presented as mean ± SD (*n* = 5). *P < 0.05 versus “Veh” treatment. ^#^*P* < 0.05 group (**D–G**). “n.s.” stands for non-statistical difference (*P* > 0.05). Experiments in this figure were repeated five times, and similar results were obtained.

The bromodomain and extraterminal domain (BET) family protein BRD4 [61] directly binds to the acetylated-histones, regulating epigenetic processes [62–64]. BRD4 is essential for expression of key oncogenic genes, including *Myc*, *cyclin D1* and *Klf4* [63, 65]. Here in patient-derived pNSCLC-1 and pNSCLC-2 cells, SKI-349 (5 μM, 12 h) treatment induced downregulation of *BRD4* mRNA and protein (Fig. 5A, B). As a result, mRNA expression of *Myc*, *cyclin D1* and *Klf4* was reduced (Fig. 5C). Next, a lentiviral BRD4-expressing construct (“oe-BRD4”) was transduced to pNSCLC-1 primary cells. It completely restored BRD4 protein expression in SKI-349-treated pNSCLC-1 cells (Fig. 5D). oe-BRD4 attenuated SKI-349-induced proliferation inhibition (EdU staining assays, Fig. 5E), cell apoptosis (by measuring TUNEL-positively nuclei ratio, Fig. 5F) and death (Fig. 5G) in pNSCLC-1 primary cells. Importantly, PF-543 single treatment or combination with ABC294640 failed to significantly affect BRD4 expression in pNSCLC-1 cells (Fig. 5H). These results implied that BRD4 downregulation could be the unique action by SKI-349, independent of SphK1/2 inhibition.

### SKI-349 administration inhibits NSCLC xenograft growth in nude mice

**Fig. 6:**
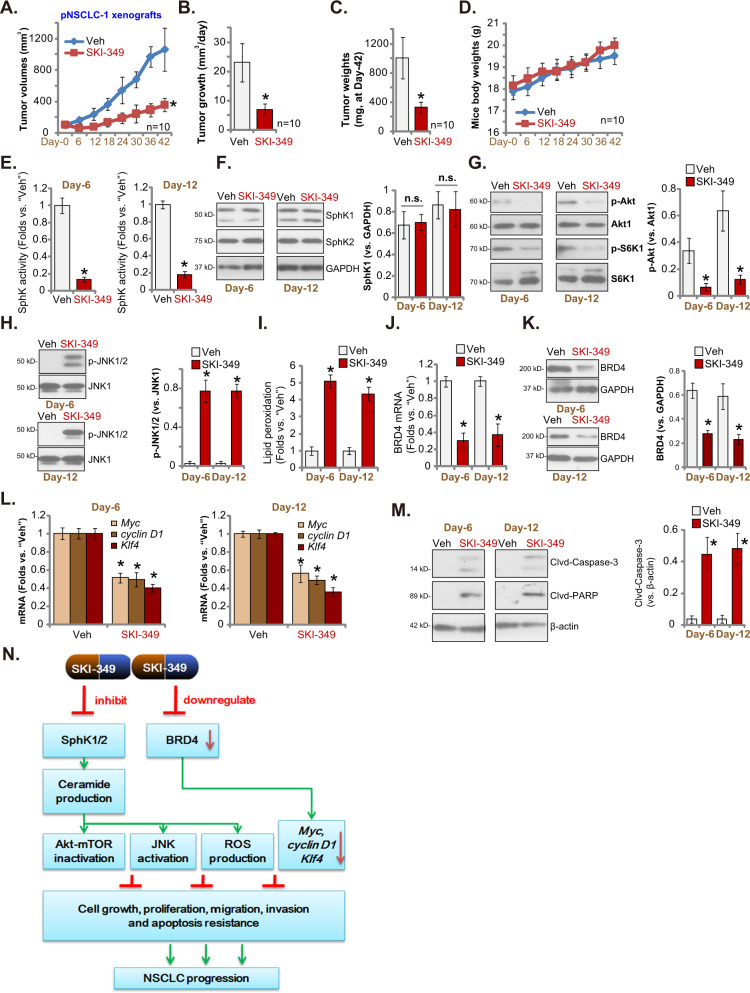
SKI-349 administration inhibits NSCLC xenograft in nude mice. The mice bearing pNSCLC-1 xenografts were subject to the applied SKI-349 administration or vehicle control treatment (“Veh”), with 10 mice per group (*n* = 10). The tumor volumes (**A**) and the mice body weights (**D**) were measured every 6 days (“Day-0” to “Day-42”); The estimated daily tumor growth (**B**) and weights of pNSCLC-1 xenografts at “Day-42” (**C**) were measured. At “Day-6” and “Day-12”, one pNSCLC-1 tumor per group were isolated, and the relative SphK activity was tested (**E**). Expression of listed genes and proteins in the described tumor tissues were tested (**F**, **G**, **H**, **J**, **K**, **L** and **M**). The relative lipid peroxidation intensity (TBAR activity, (**I**)) was examined as well. The proposed signaling pathway of the study (**N**). Data were presented as mean ± SD. *P < 0.05 versus “Veh” treatment. “n.s.” stands for non-statistical difference (*P* > 0.05).

Tumor growth curve results demonstrated that SKI-349 administration potently inhibited the growth of pNSCLC-1 xenografts in nude mice (Fig. 6A). Compared to the xenografts with vehicle control treatment, the volumes of SKI-349-treated pNSCLC-1 xenografts were significantly lower (Fig. 6A). A described formula was utilized to calculate the estimated daily tumor growth (in mm^3^ per day [21, 22]). Daily tumor growth was impeded by the applied SKI-349 administration (Fig. 6B). At “Day-42” pNSCLC-1 xenografts of the two groups were isolated and individually weighted. SKI-349-treated pNSCLC-1 tumors were significantly lighter than vehicle-treated tumors (Fig. 6C). The mice body weights, as shown in Fig. 6D, were however not significantly different between the two groups.

To analyze the potential signaling changes in vivo, at “Day-6” and “Day-12” we isolated one pNSCLC-1 xenograft per group 6 h after SKI-349/vehicle administration. A total of four xenografts were obtained and tumor tissues were freshly analyzed. As shown, the SphK activity was significantly decreased in SKI-349-treated pNSCLC-1 tumor tissues (Fig. 6E). SphK1 and SphK2 protein expression was however not significantly changed (Fig. 6F). Akt-mTOR inactivation was detected in pNSCLC-1 xenograft tissues with SKI-349 administration, as Akt and S6K1 phosphorylations were significantly decreased (Fig. 6G). Furthermore, increased JNK1/2 phosphorylation (Fig. 6H) and lipid peroxidation (TBAR activity, Fig. 6I) were detected in pNSCLC-1 xenograft tissues with SKI-349 administration. On the other hand, *BRD4* mRNA and protein levels were decreased in SKI-349-treated tumor tissues (Fig. 6J, K). Expression of BRD4-dependent mRNAs, *Myc*, *cyclin D1* and *Klf4*, was decreased as well (Fig. 6L). Contrarily, levels of cleaved-caspase-3 and cleaved-PARP were significantly increased in SKI-349-treated pNSCLC-1 xenograft tissues, implying apoptosis induction (Fig. 6M).Therefore, in line with the signaling findings in vitro, SKI-349 administration induced Akt-mTOR inactivation, JNK activation, oxidative injury and BRD4 downregulation in pNSCLC-1 xenograft tissues.

## Discussion

The results of the present study supported that targeting SphK1/2 by SKI-349 could result in profound anti-NSCLC cell activity. In primary human NSCLC cells and immortalized lines, SKI-349 potently inhibited cell proliferation, cell cycle progression, migration and viability, while provoking apoptosis. It was however non-cytotoxic to lung epithelial cells. The dual inhibitor inactivated SphK and resulted in ceramide accumulation in NSCLC cells (Fig. 6N). Significantly, there was a time-dependent response following treatment of SKI-349 treatment in NSCLC cells. SKI-349 first resulted in SphK1/2 inhibition, ceramide accumulation and signaling changes (12 h), cell cycle arrest (24 h), migration inhibition (24 h), and subsequent proliferation inhibition (48 h) and caspase/PARP activation (48 h), followed by apoptosis activation (72 h), viability reduction and cell death (72 h). In vivo, single dose SKI-349 administration robustly inhibited NSCLC xenograft growth in nude mice. Notably, SKI-349-caused anti-NSCLC cell activity was significantly more robust than the SphK1 inhibitor or plus the SphK2 specific inhibitor. Therefore, concurrent inhibition of SphK1 and SphK2 by SKI-349 resulted in robust killing of NSCLC cells.

Targeting SphK1/2, using genetic methods or pharmacologic agents, has been verified as an important strategy to inhibit NSCLC [11, 15, 16, 66, 67]. While the role of SphK1 in NSCLC has been well-studied [11–14, 66, 67], recent studies have focused on SphK2 in tumorigenesis and progression of NSCLC. Wang et al., have shown that SphK2 is overexpression in NSCLC, which is correlated with disease grade, lymph node status and NSCLC stage as well as tumor size and histology type [15]. Overexpressed SphK2 could be a valuable biomarker for prognosis and promising therapeutic target for NSCLC [15]. Yang et al., reported that ABC294640, the SphK2 inhibitor, sensitized TRAIL-induced NSCLC cell apoptosis possibly through upregulating death receptor4/5 (DR4/5) [17]. Liu et al., demonstrated that small interference RNA (siRNA)-induced silencing of SphK2 inhibited NSCLC cell proliferation and chemo-sensitized NSCLC cells to gefitinib-induced apoptosis [16].

A number of different SphK inhibitors were shown to interfere other signaling cascades in cancerous cells. SphK inhibition will induce ceramide accumulation, which can de-phosphorylate Akt by activating protein phosphatases (PP) PP1 and PP2A [56]. Moreover, SphK inhibition and ceramide accumulation could also initiate JNK activation [58–60] and ROS production [49, 51]. Sun et al., have shown that SKI-V, a SphK1 inhibitor, also inhibited Akt-mTOR activation in osteosarcoma cells [68]. Xun et al., demonstrated that the SphK2 inhibitor ABC294640 inhibited AKT-S6K1 but activated JNK signaling in colorectal cancer cells [24]. Yang et al., discovered that GDC-0349 induced Akt-mTORC1/2 blockage, SphK1 inactivation, ceramide accumulation, JNK activation and oxidative injury in NSCLC cells [69]. Yang and colleagues found that SphK2 inhibition or silencing potentiated TRAIL-induced apoptosis in NSCLC cells [17].

Due to various genetic mutations, Akt-mTOR overactivation is often detected in NSCLC, which is associated with tumorigenesis and cancer progression [70]. Indeed, Akt-mTOR overexpression and hyperactivation could be observed in over 90% of NSCLC adenocarcinoma [71–73]. Conversely, Akt-mTOR blockage would result in profound anti-NSCLC activity [70, 74, 75]. Our previous study has shown that PQR620, a novel and potent mTOR kinase blocker, efficiently inhibited NSCLC cell growth [21].

We here showed that Akt-mTOR inactivation, JNK activation and oxidative injury were detected in SKI-349-treated NSCLC cells and SKI-349-administrated pNSCLC-1 xenograft tissues. More importantly, caAkt1, the antioxidant NAC and the JNK inhibitor SP600125 all ameliorated SKI-349-induced NSCLC cell death. Therefore, alteration of these signaling cascades together could explain the superior anti-NSCLC activity by the novel SphK1/2 dual inhibitor (Fig. 6N).

BRD4 regulates epigenetic processes by associating with acetylated-histones [62–64]. BRD4 is also essential for the transcription elongation and expression of various oncogenes by associating with pTEFb (positive transcription elongation factor b) and by phosphorylating RNA polymerase II [63, 65]. Recent studies have proposed BRD4 as an important therapeutic target of NSCLC, and it is essential for the expression of Bcl-2, c-Myc, cyclin D1 and other oncogenic genes [63, 65]. Liao et al., have reported that BRD4 is overexpressed in NSCLC tissues and is correlated with histological type, lymph node metastasis, tumor stage and differentiation, and the poor prognosis [76]. Gao et al., showed that genetic silencing or pharmacological inhibition of BRD4 inhibited NSCLC cell growth possibly by downregulating eIF4E-mediated transcription [77]. BRD4 inhibition or depletion enhanced TRAIL-induced NSCLC cell apoptosis by inactivating NFκB cascade [78].

BRD4 physically associated with YAP/TAZ transcription factors, increasing expression of a number of different growth-regulating genes that are essential for the progression of cancer cells [79]. Frequent ARID1A (the AT-rich interactive domain 1A [SWI-like] gene) depletion, detected in 20% of all lung cancers, induced chromatin remodeling and glycolysis, inhibiting cell death induced by the BRD4 inhibitor JQ1 [80]. He et al., have shown that BRD4 inhibition and ARID2 depletion synergistically inhibited expression of DNA repair-related genes and induced robust cytotoxicity in cancer cells [81]. One important finding of the present study is that SKI-349 downregulated BRD4 and inhibited BRD4-dependent genes (*Myc*, *cyclin D1* and *Klf4*) in primary NSCLC cells (Fig. 6N). More importantly, SKI-349-induced NSCLC cell death was ameliorated by ectopic overexpression of BRD4. BRD4 silencing by the SphK1/2 dual inhibitor could be another reason to explain its superior anti-NSCLC cell activity.

Novel and more efficient targeted therapies are urgently needed for NSCLC. We showed that targeting SphK1/2 by SKI-349 potently inhibited NSCLC cell growth in vitro and in vivo. However, the conclusion was based on the in vitro cellular studies and animal xenograft results. The efficacy and safety of the dual inhibitor against human NSCLC warrant further characterizations under clinical studies. The underlying mechanisms of SKI-349-induced anti-NSCLC cell activity need more exploration as well.

## Supplementary information

Email copy regarding the author change

Original author exchange form

checklist form

Author contribution form

Figure S1

## Data Availability

All data are available upon request.
