# Supplementary material for: Targeting sphingosine kinase 1/2 by a novel dual inhibitor SKI-349 suppresses non-small cell lung cancer cell growth
Source: Cell Death Dis. 2022 Jul 12;13(7):602. doi: 10.1038/s41419-022-05049-4 (PMC9279331; doi:10.1038/s41419-022-05049-4)
Supplement: Supplementary file 1 — Email copy regarding the author change [file 41419_2022_5049_MOESM1_ESM.pdf]

回复：CDDIS-22-0741RR Initial Quality Check

精简信息

发件人：杨仪<yaungyi@163.com>

收件人：xuweihua2208<xuweihua2208@suda.edu.cn>

时　间：2022年06月24日 09:04 (星期五)

Confirmed

---- 回复的原邮件 ----

发件人	徐卫华<xuweihua2208@suda.edu.cn>
日期	2022年06月23日 08:12
收件人	yaungyi@163.com<yaungyi@163.com>、1300245411@qq.com<1300245411@qq.com>、jiangkanqiu@163.com<jiangkanqiu@163.com>、shenmingjing0707@126.com<shenmingjing0707@126.com>、xuweihua2208@suda.edu.cn<xuweihua2208@suda.edu.cn>、marvel_j@163.com<marvel_j@163.com>、1295167994@qq.com<1295167994@qq.com>
主題	Fw:CDDIS-22-0741RR Initial Quality Check

Dear co-authors

For the paper CDDIS-22-0741RR entitled "***Targeting sphingosine kinase 1/2 by a novel dual inhibitor SKI-349 suppresses non-small cell lung cancer cell growth***" back to ***Cell Death Disease.***

Author "Li Ou" isolated and verified primary human NSCLC cells/epithelial cells and performed mitochondrial functional assays and apoptosis experiments in the primary cells. "Li Ou" also performed revised experiments. Author "Li Ou" was accidently omitted in the original submission and was therefore added in the revised MS. Thanks.

RE：Fw:CDDIS-22-0741RR Initial Quality Check

优化阅读 | 精简信息

发件人：薛宇航<1300245411@qq.com>

收件人：yaungyi<yaungyi@163.com> jiangkanqiu<jiangkanqiu@163.com> shenmingjing0707<shenmingjing0707@126.com> xuweihua2208<xuweihua2208@suda.edu.cn> marvel_j<marvel_j@163.com> ↓还有1个联系人 保存所有收件人

时　间：2022年06月23日 10:14 (星期四)

Confirmed

- 隐藏引用文字 -

------------------ 原始邮件 ------------------

发件人: "徐卫华" <xuweihua2208@suda.edu.cn>;
发送时间: 2022年6月23日(星期四) 上午8:12
收件人: "yaungyi"<yaungyi@163.com>;"薛宇航"<1300245411@qq.com>;"jiangkanqiu"<jiangkanqiu@163.com>;"shenmingjing0707"<shenmingjing0707@126.com>;"xuweihua2208"<xuweihua2208@suda.edu.cn>;"marvel_j"<marvel_j@163.com>;"1295167994"<1295167994@qq.com>;
主题: Fw:CDDIS-22-0741RR Initial Quality Check

Dear co-authors

For the paper CDDIS-22-0741RR entitled "***Targeting sphingosine kinase 1/2 by a novel dual inhibitor SKI-349 suppresses non-small cell lung cancer cell growth***" back to ***Cell Death Disease.***

Author "Li Ou" isolated and verified primary human NSCLC cells/epithelial cells and performed mitochondrial functional assays and apoptosis experiments in the primary cells. "Li Ou" also performed revised experiments. Author "Li Ou" was accidently omitted in the original submission and was therefore added in the revised MS. Thanks.

Please confirm.

Wei-hua Xu

Re:Fw:CDDIS-22-0741RR Initial Quality Check

优化阅读 | 精简信息

发件人：jingjing lu<marvel_j@163.com>

收件人：徐卫华<xuweihua2208@suda.edu.cn>

抄送人：yaungyi<yaungyi@163.com> 1300245411<1300245411@qq.com> jiangkanqiu<jiangkanqiu@163.com> shenmingjing0707<shenmingjing0707@126.com> xuweihua2208<xuweihua2208@suda.edu.cn> ↓还有1个联系人 保存所有抄送人

时　间：2022年06月23日 09:26 (星期四)

Confirmed

At 2022-06-23 08:12:12, "徐卫华" <xuweihua2208@suda.edu.cn> wrote:

- 隐藏引用文字 -

Dear co-authors

For the paper CDDIS-22-0741RR entitled "***Targeting sphingosine kinase 1/2 by a novel dual inhibitor SKI-349 suppresses non-small cell lung cancer cell growth***" back to ***Cell Death Disease.***

Author "Li Ou" isolated and verified primary human NSCLC cells/epithelial cells and performed mitochondrial functional assays and apoptosis experiments in the primary cells. "Li Ou" also performed revised experiments. Author "Li Ou" was accidently omitted in the original submission and was therefore added in the revised MS. Thanks.

Please confirm.

Wei-hua Xu

Re: CDDIS-22-0741RR Initial Quality Check

发件人：Jiang Kanqiu<jiangkanqiu@163.com>

收件人：徐卫华<xuweihua2208@suda.edu.cn>

时　间：2022年06月23日 08:51 (星期四)

Confirmed

Kanqiu Jiang

邮箱：jiangkanqiu@163.com

---- Replied Message ----

From	徐卫华<xuweihua2208@suda.edu.cn>
Date	06/23/2022 08:12
To	yaungyi<yaungyi@163.com>,<br>1300245411<1300245411@qq.com>,<br>jiangkanqiu<jiangkanqiu@163.com>,<br>shenmingjing0707<shenmingjing0707@126.com>,<br>xuweihua2208<xuweihua2208@suda.edu.cn>,<br>marvel_j<marvel_j@163.com>,<br>1295167994<1295167994@qq.com>
Subject	Fw:CDDIS-22-0741RR Initial Quality Check

Dear co-authors

For the paper CDDIS-22-0741RR entitled "***Targeting sphingosine kinase 1/2 by a novel dual inhibitor SKI-349 suppresses non-small cell lung cancer cell growth***" back to ***Cell Death Disease.***

回复：CDDIS-22-0741RR Initial Quality Check

发件人：沈明敬<shenmingjing0707@126.com>

收件人：xuweihua2208<xuweihua2208@suda.edu.cn>

时　间：2022年06月23日 08:39 (星期四)

confirmed

---- 回复的原邮件 ----

发件人	徐卫华<xuweihua2208@suda.edu.cn>
日期	2022年06月23日 08:12
收件人	yaungyi@163.com<yaungyi@163.com>、1300245411@qq.com<1300245411@qq.com>、jiangkanqiu@163.com<jiangkanqiu@163.com>、shenmingjing0707@126.com<shenmingjing0707@126.com>、xuweihua2208@suda.edu.cn<xuweihua2208@suda.edu.cn>、marvel_j@163.com<marvel_j@163.com>、1295167994@qq.com<1295167994@qq.com>
主题	Fw:CDDIS-22-0741RR Initial Quality Check

Dear co-authors

For the paper CDDIS-22-0741RR entitled "***Targeting sphingosine kinase 1/2 by a novel dual inhibitor SKI-349 suppresses non-small cell lung cancer cell growth***" back to ***Cell Death Disease.***

Author "Li Ou" isolated and verified primary human NSCLC cells/epithelial cells and performed mitochondrial functional assays and apoptosis experiments in the primary cells. "Li Ou" also performed revised experiments. Author "Li Ou" was accidently omitted in the original submission and was therefore added in the revised MS. Thanks.

Please confirm.

Wei-hua Xu

<< 返回 | 回复 | 回复全部 | 转发 | 删除 | 举报 | 标记为 | 移动到 | 更多

Re: Fw:CDDIS-22-0741RR Initial Quality Check

发件人：欧小妮<1295167994@qq.com>

收件人：徐卫华<xuweihua2208@suda.edu.cn> yaungyi<yaungyi@163.com> 1300245411<1300245411@qq.com> jiangkanqiu<jiangkanqiu@163.com> shenmingjing0707<shenmingjing0707@126.com> ↓还有1个联系人 保存所有收件人

时　间：2022年06月23日 08:16 (星期四)

Confirmed

发自我的iPhone

------------------ Original ------------------

From: 徐卫华 <xuweihua2208@suda.edu.cn>
Date: Thu,Jun 23,2022 8:12 AM
To: yaungyi <yaungyi@163.com>, 1300245411 <1300245411@qq.com>, jiangkanqiu <jiangkanqiu@163.com>, shenmingjing0707 <shenmingjing0707@126.com>, xuweihua2208 <xuweihua2208@suda.edu.cn>, marvel_j <marvel_j@163.com>, 1295167994 <1295167994@qq.com>
Subject: Re: Fw:CDDIS-22-0741RR Initial Quality Check

Dear co-authors

For the paper CDDIS-22-0741RR entitled "***Targeting sphingosine kinase 1/2 by a novel dual inhibitor SKI-349 suppresses non-small cell lung cancer cell growth***" back to ***Cell Death Disease.***

Author "Li Ou" isolated and verified primary human NSCLC cells/epithelial cells and performed mitochondrial functional assays and apoptosis experiments in the primary cells. "Li Ou" also performed revised experiments. Author "Li Ou" was accidently omitted in the original submission and was therefore added in the revised MS. Thanks.

Please confirm.
