# Supplementary material for: Targeting sphingosine kinase 1/2 by a novel dual inhibitor SKI-349 suppresses non-small cell lung cancer cell growth
Source: Cell Death Dis. 2022 Jul 12;13(7):602. doi: 10.1038/s41419-022-05049-4 (PMC9279331; doi:10.1038/s41419-022-05049-4)
Supplement: Supplementary file 4 — Author contribution form [file 41419_2022_5049_MOESM4_ESM.pdf]

DECLARATION OF CONTRIBUTIONS TO ARTICLE

ADMC

Manuscript Number:

Journal Name: *Cell Death & Disease* (the 'Journal')

Proposed Title of the Contribution:

Targeting sphingosine kinase 1/2 by a novel dual inhibitor SKI-349 suppresses non-small cell lung cancer cell growth (the 'Contribution)

Author(s):

Yuhang Xue, Kanqiu Jiang, Li Ou, Mingjing Shen, Yi Yang, Jingjing Lu, Weihua Xu (the 'Authors')

For all *CDDis* articles, each person named as an author in the published version must be able to show he or she has contributed substantially to the article.

Authorship credit should be based on 1) substantial contributions to conception and design, acquisition of data, or analysis and interpretation of data; 2) drafting the article or revising it critically for important intellectual content; and 3) final approval of the version to be published. Authors should meet conditions 1, 2 and 3.

Any person who cannot be shown to have made a substantial contribution to the article cannot be listed as an author in the final version. The name of any person who is deemed to have made a minor contribution can, however, appear in the Acknowledgments section of the article.

Please complete the table below to indicate the contributions of all named authors to the manuscript.

Author Full Name:	Specification of Contribution to the Manuscript:
Yuhang Xue	Functional assays in NSCLC cells and normal cells, establishing NSCLC xenografts, testing signalings and cell death/apoptosis, drafting the article and revising it, with final approval of the version submitted to the journal.
Kanqiu Jiang	Functional assays in NSCLC cells and normal cells, establishing NSCLC xenografts, testing signalings and cell death/apoptosis, drafting the article and revising it, with final approval of the version submitted to the journal.
Li Ou	Functional assays in NSCLC cells and normal cells, establishing NSCLC xenografts, testing signalings and cell death/apoptosis, drafting the article and revising it, with final approval of the version submitted to the journal.
Mingjing Shen	Functional assays in NSCLC cells and normal cells, establishing NSCLC xenografts, testing signalings and cell death/apoptosis, drafting the article and revising it, with final approval of the version submitted to the journal.
Yi Yang	Study conception and design, and data analysis, Figure organization, involved in drafting the article and revising it critically for important intellectual content, and with final approval of the version submitted to the journal.
Jingjing Lu	Study conception and design, and data analysis, Figure organization, involved in drafting the article and revising it critically for important intellectual content, and with final approval of the version submitted to the journal.
Weihua Xu	Study conception and design, and data analysis, Figure organization, involved in drafting the article and revising it critically for important intellectual content, and with final approval of the version submitted to the journal.

Please complete the table below to indicate the contributions of all named authors to the figures.

Figure 1:

Yuhang Xue, Kanqiu Jiang, Li Ou, Mingjing Shen, Yi Yang, Jingjing Lu, Weihua Xu

Figure 2:

Yuhang Xue, Kanqiu Jiang, Li Ou, Mingjing Shen, Yi Yang, Jingjing Lu, Weihua Xu

Figure 3:

Yuhang Xue, Kanqiu Jiang, Li Ou, Mingjing Shen, Yi Yang, Jingjing Lu, Weihua Xu

Figure 4:

Yuhang Xue, Kanqiu Jiang, Li Ou, Mingjing Shen, Yi Yang, Jingjing Lu, Weihua Xu

Figure 5:

Yuhang Xue, Kanqiu Jiang, Li Ou, Mingjing Shen, Yi Yang, Jingjing Lu, Weihua Xu

Figure 6:

Yuhang Xue, Kanqiu Jiang, Li Ou, Mingjing Shen, Yi Yang, Jingjing Lu, Weihua Xu

Signed for and on behalf of the Author(s):	Print Name:	Date:
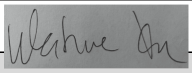	Weihua Xu	2022-05-31
