# Supplementary material for: Targeting sphingosine kinase 1/2 by a novel dual inhibitor SKI-349 suppresses non-small cell lung cancer cell growth
Source: Cell Death Dis. 2022 Jul 12;13(7):602. doi: 10.1038/s41419-022-05049-4 (PMC9279331; doi:10.1038/s41419-022-05049-4)

Figure S1.The uncropped blotting images of the study.

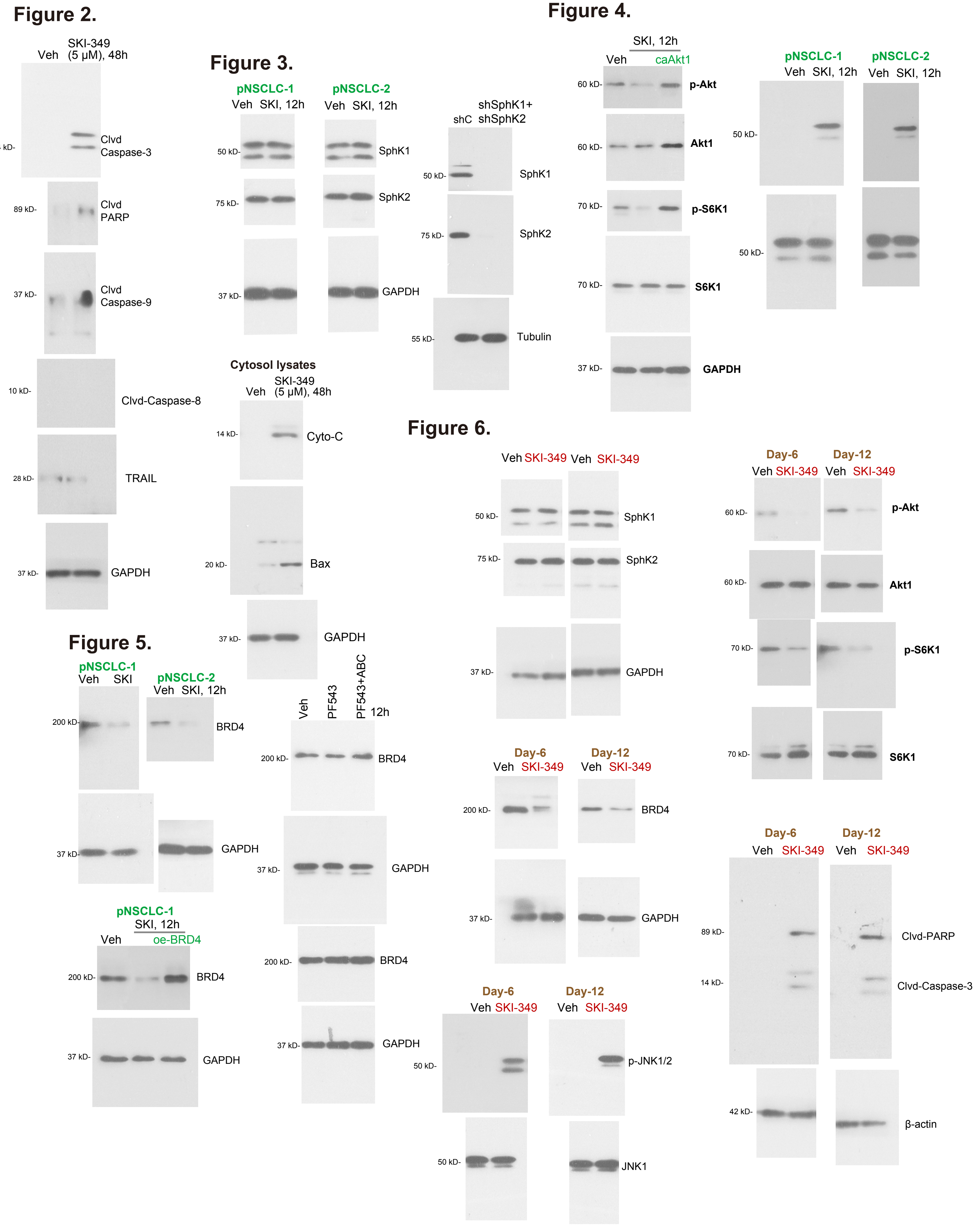

Supplement: Supplementary file 5 — Figure S1 [file 41419_2022_5049_MOESM5_ESM.pdf]
